# Bayesian intravoxel incoherent motion parameter mapping in the human heart

**DOI:** 10.1186/s12968-017-0391-1

**Published:** 2017-11-06

**Authors:** Georg R. Spinner, Constantin von Deuster, Kerem C. Tezcan, Christian T. Stoeck, Sebastian Kozerke

**Affiliations:** 1 Institute for Biomedical Engineering, University and ETH Zurich, Gloriastrasse 35, 8092 Zurich, Switzerland; 2 Computer Vision Laboratory, ETH Zurich, Sternwartstrasse 7, 8092 Zurich, Switzerland

**Keywords:** Cardiac diffusion imaging, motion-compensated diffusion weighted spin-echo, intravoxel incoherent motion, Bayesian inference, perfusion

## Abstract

**Background:**

Intravoxel incoherent motion (IVIM) imaging of diffusion and perfusion in the heart suffers from high parameter estimation error. The purpose of this work is to improve cardiac IVIM parameter mapping using Bayesian inference.

**Methods:**

A second-order motion-compensated diffusion weighted spin-echo sequence with navigator-based slice tracking was implemented to collect cardiac IVIM data in early systole in eight healthy subjects on a clinical 1.5 T CMR system. IVIM data were encoded along six gradient optimized directions with b-values of 0–300 s/mm^2^. Subjects were scanned twice in two scan sessions one week apart to assess intra-subject reproducibility. Bayesian shrinkage prior (BSP) inference was implemented to determine IVIM parameters (diffusion *D*, perfusion fraction *F* and pseudo-diffusion D*). Results were compared to least-squares (LSQ) parameter estimation. Signal-to-noise ratio (SNR) requirements for a given fitting error were assessed for the two methods using simulated data. Reproducibility analysis of parameter estimation in-vivo using BSP and LSQ was performed.

**Results:**

BSP resulted in reduced SNR requirements when compared to LSQ in simulations. In-vivo, BSP analysis yielded IVIM parameter maps with smaller intra-myocardial variability and higher estimation certainty relative to LSQ. Mean IVIM parameter estimates in eight healthy subjects were (LSQ/BSP): 1.63 ± 0.28/1.51 ± 0.14·10^−3^ mm^2^/s for *D*, 13.13 ± 19.81/13.11 ± 5.95% for *F* and 201.45 ± 313.23/13.11 ± 14.53·10^−3^ mm^2^/s for *D* ^∗^. Parameter variation across all volunteers and measurements was lower with BSP compared to LSQ (coefficient of variation BSP vs. LSQ: 9% vs. 17% for *D*, 45% vs. 151% for *F* and 111% vs. 155% for *D* ^∗^). In addition, reproducibility of the IVIM parameter estimates was higher with BSP compared to LSQ (Bland-Altman coefficients of repeatability BSP vs. LSQ: 0.21 vs. 0.26·10^−3^ mm^2^/s for *D*, 5.55 vs. 6.91% for *F* and 15.06 vs. 422.80·10^−3^ mm^2^/s for D*).

**Conclusion:**

Robust free-breathing cardiac IVIM data acquisition in early systole is possible with the proposed method. BSP analysis yields improved IVIM parameter maps relative to conventional LSQ fitting with fewer outliers, improved estimation certainty and higher reproducibility. IVIM parameter mapping holds promise for myocardial perfusion measurements without the need for contrast agents.

## Background

Cardiovascular magnetic resonance (CMR) diffusion weighted imaging relies on signal attenuation due to random motion of water molecules in the presence of diffusion encoding gradients. Additionally, microvascular perfusion can contribute to the signal loss as described by the intravoxel incoherent motion (IVIM) model [1, 2]. According to Le Bihan et al. [3, 4], perfusion can be modeled as pseudo diffusion on a macroscopic scale, assuming random orientation of microvasculature in the capillary network. Consequently, the signal intensity can be described by a bi-exponential signal decay as a function of the diffusion encoding strength (b-value). As the IVIM method is an endogenous contrast technique, its application is particularly suited to obtain a tissue perfusion surrogate where contrast agent administration is contraindicated. In recent years, this technique has gained significant momentum with successful applications in various body parts [5–9]. Beyond body and brain applications, IVIM of the in-vivo human heart has also been demonstrated [10, 11].

Cardiac IVIM may allow to delineate infarcted and ischemic areas showing good agreement with late-gadolinium enhanced imaging [12, 13]. Moreover, IVIM may enable the assessment of chronic and acute ischemia [14] as well as conditions related to microvascular obstruction of the myocardium [15].

Despite recent progress, in-vivo cardiac diffusion weighted imaging still remains challenging due to cardiac and respiratory motion. Additionally, low signal-to-noise ratio (SNR) and long scan times are major impediments to a wider acceptance in a clinical setting. Motion induced signal loss in spin-echo (SE) based cardiac diffusion weighted imaging has been addressed by first-order motion-compensated diffusion gradient designs in conjunction with careful cardiac trigger delay selection [16] and more recently by second-order motion compensation [17–19]. Initial results of the application of second-order motion compensation for IVIM acquisitions during systole have previously been presented in a porcine model [14]. For diastolic imaging, time-shifted triggering and dedicated post processing using principal component analysis (PCA) filtering in combination with temporal maximum intensity projection (PCATMIP) has been proposed [20–23].

Experimentally, cardiac IVIM parameters were initially reported for the in-vivo canine heart by Callot et al. [24]. The measured diffusion weighted signal agreed well with the bi-exponential IVIM model with reduced signal decay in the absence of perfusion post-mortem [14].

IVIM parameter maps of various organs such as brain or heart [20, 25] are typically of noisy appearance. Due to the non-linearity and bad conditioning of the regression problem, the perfusion related parameters are estimated with considerable error at typical SNR values as shown in [26, 27]. Besides modifying the data acquisition protocol to obtain higher SNR at the expense of lower spatial resolution and/or longer scan time, group analysis of longitudinal data of individuals incorporating both intra- and inter-subject variations [28] or regional smoothing [29] have been proposed. These approaches are, however, limited by the necessity of repeated measurements across multiple independent subjects or loss of spatial resolution and increased partial-voluming, respectively.

To address the SNR limitation of IVIM analysis, a hierarchical Bayesian data analysis framework has been presented by Orton et al. [30] and demonstrated for liver application. Using this approach, information across the region-of-interest is taken into account for voxelwise parameter inference. Parameter estimation is performed using a posterior distribution combining data likelihood and a hierarchical prior. This combination enables effective denoising of parameter maps with reduced parameter estimation error.

The objective of the present work was to implement and assess Bayesian shrinkage prior (BSP) inference for IVIM parameter mapping of the in-vivo human heart and compare its performance to segmented least-squares (LSQ) fitting.

## Theory

### Intravoxel incoherent motion

The IVIM model [1] in Eq. (1) describes signal magnitude of diffusion weighted images as bi-exponential decay. In addition to diffusion induced signal attenuation, a second compartment of perfusion induced pseudo-diffusion is taken into account:

1 $$ S(b)={S}_0\left[F\cdot \exp \left(-{bD}^{\ast}\right)+\left(1-F\right)\cdot \exp \left(- bD\right)\right] $$

where *S*(*b*) describes the measured signal as a function of b-value, *S*_0_ the signal without diffusion weighting (*b*=0 s/mm^2^), *D* the diffusion constant, *F* the perfusion fraction and *D* ^∗^ the pseudo-diffusion constant. Note that capital *F* is used for the perfusion fraction to be consistent with the notation of Orton et al. [30].

### Least-squares fitting

For LSQ fitting, a segmented approach [6] is implemented assuming the contribution of the perfusion to reach a maximum of *F*/(1 − *F*) at *b*=0 s/mm^2^ and to drop to negligible values for b-values *b* ≫ *b*_*Split*_. In practice, high b-values (*b* ≥ *b*_*Split*_=200 s/mm^2^) are fitted to a mono-exponential diffusion-only model:

2 $$ S(b)\approx {S}_0\cdot \left(1-F\right)\cdot \exp \left(- bD\right)={S}_{int}\cdot \exp \left(- bD\right) $$

If a non-diffusion weighted image *S*_0_ is not available, the intercept *S*_*int*_ does not allow for a direct calculation of the perfusion fraction *F* as described in [6], but *S*_*int*_ = *S*_0_ ∙ (1 − *F*) enables to eliminate *S*_0_ in the bi-exponential model. In the second step of the segmented regression, the perfusion related parameters *F* and *D* ^∗^ are estimated using the predetermined diffusion coefficient *D* and the mono-exponential intercept *S*_*int*_ while taking into account all considered b-values. By substituting *S*_0_ in Eq. (1), the signal model reads accordingly:

3 $$ {\displaystyle \begin{array}{c}S(b)={S}_0\left[F\cdot \exp \left(-{bD}^{\ast}\right)+\left(1-F\right)\cdot \exp \left(- bD\right)\right]\\ {}={S}_{\mathrm{int}}\left[\frac{F}{1-F}\cdot \exp \left(-{bD}^{\ast}\right)+\exp \left(- bD\right)\right]\end{array}} $$

The nonlinear regression is implemented using an interior-point algorithm in Matlab (Mathworks, Natick, Massachusetts, USA) and constrained by an inequality together with box constraints as in [30]:

4 $$ {\displaystyle \begin{array}{c}D\le {D}^{\ast}\\ {}4.5\cdot {10}^{-5}\le D\le 1.8\cdot {10}^{-2}{mm}^2/s\\ {}0.0005\le F\le 0.9995\\ {}3.4\cdot {10}^{-4}\le {D}^{\ast}\le 1.0\cdot {10}^{-1}{mm}^2/s\end{array}} $$

### Bayesian shrinkage prior inference

For Bayesian inference as presented in [30], a marginalized data likelihood is used along with a multivariate Gaussian prior combined with Jeffrey’s prior [31]. The approach is implemented using a Markov chain Monte Carlo (MCMC) method as described in the Appendix 1.

## Methods

### Computer simulations

IVIM parameter ranges were simulated according to values reported for the in-vivo heart [14, 20]. The diffusion coefficient was set to *D*=1.5·10^−3^ mm^2^/s, while three perfusion regimes (low, intermediate, high) were considered (*F*/*D* ^∗^=10/10, 15/15, 20/20 %/10^−3^ mm^2^/s). The simulated SNRs ranged from 10 to 100 in steps of 10 and from 100 to 200 in steps of 25. Gaussian distributed noise was added followed by magnitude detection to yield Rician distributed noise mimicking the noise distribution of CMR magnitude images. A single Monte Carlo simulation run consisted of 1,000 IVIM data sets with b-values as used in the in-vivo part of this study: 20 to 100 s/mm^2^ in steps of 20 s/mm^2^, 125 to 200 s/mm^2^ in steps of 25 s/mm^2^, 250 and 300 s/mm^2^. Both bias $\left|\left\langle \widehat{p}\right\rangle -{p}_{Ref}\right|/{p}_{Ref}$ and variation ${\widehat{\sigma}}_p/{p}_{Ref}$ with *p* = *D*, *F* and *D* ^∗^, *p*_Ref_ the simulated parameter, $\left\langle \widehat{p}\right\rangle$ the mean estimate and ${\widehat{\sigma}}_p$ the standard deviation of the estimated parameters were calculated and are reported as relative errors. The simulation was repeated and resulting parameter estimation errors were averaged 100 times.

### In-vivo measurements

**Fig. 1:**
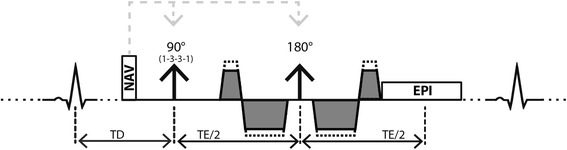
Sequence diagram. Spin-echo acquisition with second-order motion-compensated diffusion encoding gradients. Fat suppression is achieved by a 1–3–3-1 binomial spatial spectral excitation pulse. The excitation slab is tilted orthogonally with respect to the 180° pulse to allow for reduced field-of-view imaging. Prior to the diffusion weighting, a 1D–navigator pencil beam is used for automatic slice tracking by shifting the excitation and echo pulses. Various b-values are achieved by keeping timing constant while varying gradient strengths (dotted gradient trapezoids)

Second-order motion-compensated SE diffusion weighted imaging [17, 18] was implemented on a 1.5 T CMR system (Achieva, Philips Healthcare, Best, The Netherlands), see Fig. 1. Signal was received with a 5 channel cardiac receiver array. Written informed consent was obtained from all subjects prior to imaging. The study protocol was approved by the ethics committee of the Canton of Zurich. Consent included imaging as well as publication of anonymized data.

Data were acquired in eight healthy subjects without history of cardiac disease (6 female, 2 male, weight 64 ± 8 kg, age 26 ± 4 years, heart rate 64 ± 9 beats/min, min/max heart rates: 51/81 beats/min) on two separate occasions. Prior to diffusion imaging, cine data with a temporal resolution of 10 ms were acquired in two chamber and short axis view orientations. Using the cine images, systolic quiescent time points were determined visually on a per subject basis.

CMR diffusion weighted imaging was performed during free-breathing in short-axis view orientation using single-shot EPI read-out with the reduced field-of-view (FOV) technique local-look (LoLo) [32]. Slice tracking to account for breathing motion was controlled by a respiratory 1D navigator pencil beam placed on the right hemi diaphragm, accepting all data. A 1–3–3-1 binomial spectral-spatial excitation pulse for fat suppression [33] was employed. Images were acquired with in-plane resolution: 2.4 × 2.4 mm^2^, slice thickness: 10 mm, one mid-ventricular slice, FOV: 230 × 105 mm^2^, acquired k-space lines: 43, TR/TE: 2 R-R/73 ms, flip angle: 81 ± 1° (heart rate dependent Ernst angle [34]), 8 signal averages and 6 vendor gradient optimized diffusion encoding directions. The applied diffusion encoding strengths included the values described in the previous sections (20 – 100 s/mm^2^ in steps of 20 s/mm^2^, 125 – 200 s/mm^2^ in steps of 25 s/mm^2^, 250 and 300 s/mm^2^) together with 0 s/mm^2^. The trigger delay for the SE sequence was set to 25% peak systolic contraction [17] with a mean trigger delay of 78 ± 3 ms. Acquisition of the 8 signal averages for each diffusion encoding strength and direction was equally distributed along the measurement. Total scan time was about 18 min at a heart rate of 60 beats per minute.

In-vivo SNR measurements were performed in each subject. To measure noise, the scans were repeated with radio-frequency and gradient pulses switched off. Sufficient time (>10 s) was allowed between image and noise acquisition to ensure complete signal decay. SNR was determined for each voxel as described in [35].

Imaging in each subject was repeated in consecutive sessions separated by one week to assess intra-subject reproducibility.

In addition, diffusion data in an animal model of myocardial infarction was evaluated; see Appendix 2 for further details.

### Data post-processing

**Fig. 2:**
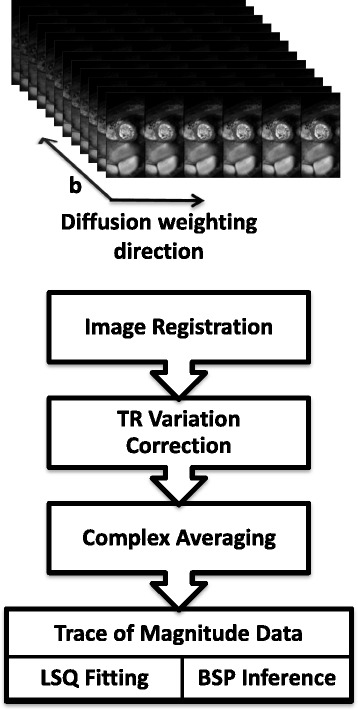
Post-processing workflow. Acquired data consists of six diffusion encoding directions, twelve diffusion encoding strengths (b-values) and eight averages. Example magnitudes are displayed at the top. The lower post-processing workflow diagram summarizes consecutive data handling steps in every volunteer. After image reconstruction, image registration is performed to compensate for residual geometric inconsistencies. Heart rate variations and hence signal fluctuations due to TR variations are compensated using recorded ECG signals. Trace data is generated after complex averaging. This data is used for IVIM parameter estimation employing both LSQ and BSP regression

For in-vivo IVIM parameter mapping, images were first registered using a dedicated groupwise image registration method [36] employing total variation displacement regularization and a PCA-based image similarity metric [37] to correct for in-plane residual geometric inconsistencies. Afterwards, complex averaging [38] of the signal averages was performed. The IVIM parameters of both regression methods (LSQ and BSP) and SNR were determined upon manual masking the left ventricular myocardium. The same segmentation was used for both regression methods (LSQ and BSP). To avoid partial volume effects, voxels at the epi- and endocardial borders were excluded during the segmentation process and all voxels within the segmented region-of-interest were used for further analysis. The image magnitudes were corrected for heart rate variations using recorded R-R intervals and published T_1_ values of the myocardium [39]. Figure 2 summarizes all post processing steps. IVIM analysis was performed on data with *b* ≥ 20 s/mm^2^ to suppress artifacts from blood flow while mean SNR was determined on *b*=0 s/mm^2^ images.

For BSP inference, the total number of MCMC samples was set to *N*_*s*_=20,000. A “burn-in” period of 10,000 (discarded) samples was used before actual sampling. The Markov chains were initialized with LSQ estimates of the IVIM parameters. Note that the Markov chains can be started from arbitrary starting values, however a starting point close to the actual parameter estimates shortens the burn-in phase and hence saves computation time. Further details of the estimation method can be found in the Appendix of Orton et al. [30]. A vectorized approach of the referenced procedure was implemented in Matlab (Mathworks) and run on standard PC hardware (2.9 GHz, 16 GB RAM).

### Reproducibility analysis

In order to assess reproducibility of two consecutive scans, Bland-Altman analysis was performed and the coefficient of variability was calculated for both scan sessions.

## Results

### Computer simulations

**Fig. 3:**
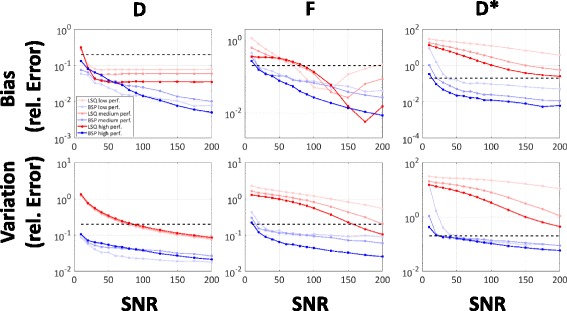
Simulation. Accuracy (bias) and precision (variation) errors of LSQ and BSP are determined from SNR 10 to 200 on simulated data for three perfusion regimes. Dashed black lines indicate 20% error

In Fig. 3, relative errors of *D*, *F* and *D* ^∗^ for BSP versus LSQ as a function of SNR are reported for a Monte Carlo simulation. Both methods show overall decreasing errors for increasing SNR.

The bias of *D* with both LSQ and BSP is reduced to below 20% for all perfusion scenarios at SNR ≥ 20, with the bias of LSQ remaining between 3 and 10% even at high SNRs. The variation of *D* with BSP is consistently lower over the entire SNR range compared to LSQ. It drops below 20% error at a SNR of approx. 90 for LSQ, but remains below 20% already for the lowest simulated SNR of 10 for BSP. Estimation of the perfusion fraction *F* yields lower bias with BSP relative to LSQ between an SNR of 20 to 90–150 depending on the perfusion regime tested. LSQ shows an increase in bias for SNR ≥ 125–175. The variation of *F* with BSP is consistently lower compared to LSQ over the entire SNR range and perfusion regimes. The relative error is below 20% for SNR ≥ 30 if BSP is used for inference. Depending on the perfusion regime simulated, the error using LSQ remains above that threshold except for the high perfusion regime at a SNR ≥ 175. The SNR dependency of *D* ^∗^ shows consistently lower bias and variation for BSP relative to LSQ for SNR ≥ 20. For LSQ, bias remains above 20% error for an SNR of 200 for all perfusion regimes. Variation of *D* ^∗^ with LSQ remains also above 20% even at an SNR of 200 for all perfusion regimes while bias and variation with BSP are bound to below 20% for SNR values above 40 and 60, respectively. Based on the simulation, an overall minimum SNR of 30–60 depending on the perfusion regime is identified for BSP to determine *D*, *F*, and *D* ^∗^ within 20% bias and variation. The LSQ method exhibits errors above the mentioned threshold even at a SNR of 200.

### In-vivo measurements

**Fig. 4:**
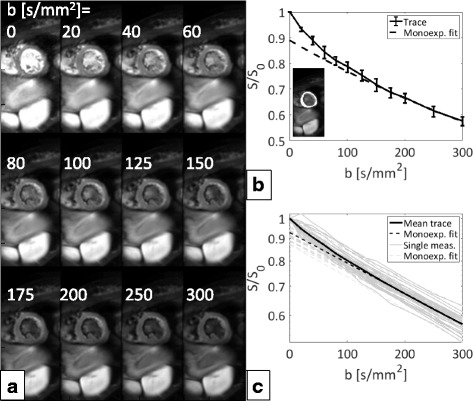
In-vivo data. Example averaged co-registered magnitude trace dataset showing all recorded b-values (**a**), averaged magnitude signal across the region-of-interest (highlighted in the small inset) of the same volunteer with error bars showing variations among diffusion encoding directions together with a mono-exponential fit for b ≥ 200 mm^2^/s to distinguish the perfusion contribution (**b**). Mean magnitude signals across all volunteers and repetitions together with the mean over all measurements and corresponding mono-exponential fits (**c**). Deviations from a purely mono-exponential model are discernable for small b-values (b < 150 mm^2^/s) in **b** and **c**. Note that the plots **b** and **c** have logarithmic y-axes

The in-vivo SNR measured without diffusion weighting was 19 ± 3 for one signal average, resulting in an SNR of approximately 54 for averaged data. Figure 4 shows example in-vivo magnitude images of all b-values. The bright blood pool signal in the center of the image is dephased with increasing diffusion weighting. Example trace magnitude signals averaged across the region-of-interest are displayed. In addition, trace signals from all volunteers and repetitions are plotted.

**Fig. 5:**
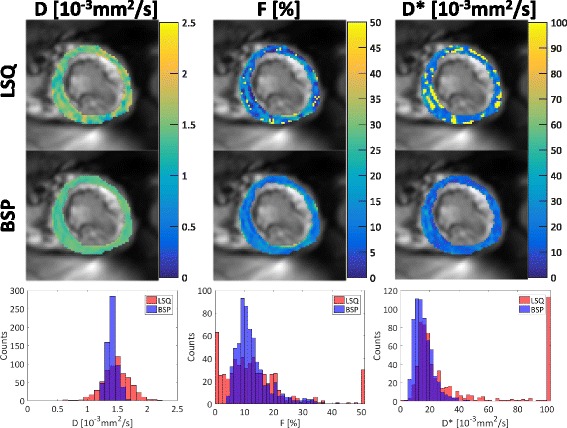
IVIM parameter maps with corresponding histograms**.** Spatial variation of the parameters is reduced for the Bayesian approach (BSP) relative to least-squares (LSQ). Histograms show corresponding narrower distributions for BSP versus LSQ. Note that local variations in *F* are preserved with the BSP method. Outliers are greatly reduced with BSP for *F* and *D* ^∗^

In Fig. 5, example IVIM parameter maps computed with LSQ and BSP along with corresponding histograms are shown. While LSQ maps exhibit spatial noise and patch-like structures, BSP yields a more uniform distribution in the myocardium which is reflected in narrower distributions of *D*, *F* and *D* ^∗^. Of note, LSQ resulted in a high number of voxels in which the estimated IVIM parameters reached or were close to the box constraints.

**Fig. 6:**
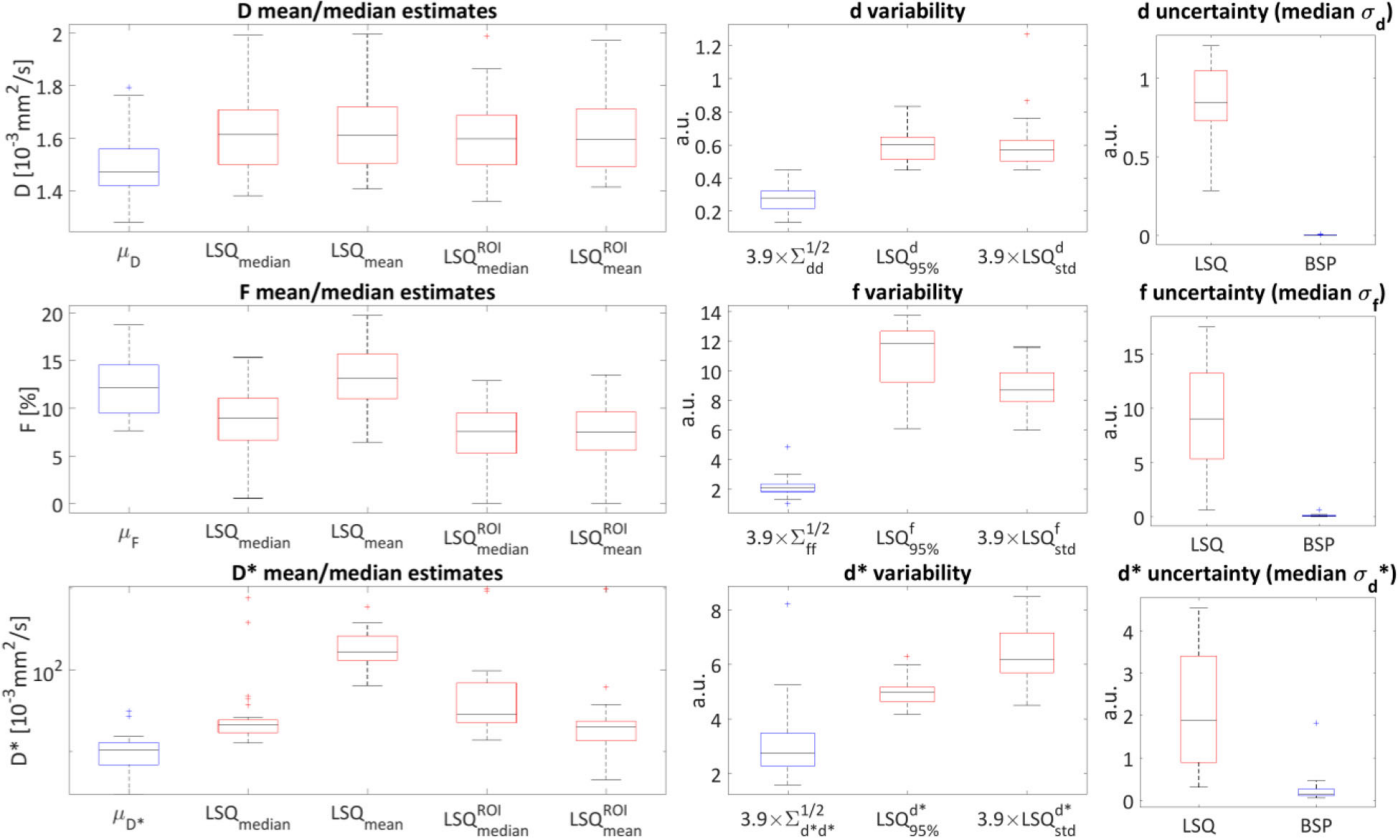
Parameter regression Box-and-Whisker plots. Red boxes represent LSQ derived values; blue boxes represent BSP derived estimates across all measurements (volunteers and repetitions). Left column: BSP prior mean values *(μ*_*p*_, *p* = *D*, *F* and *D* ^∗^), means/medians of LSQ pixel-wise (*LSQ*_*mean*_ and *LSQ*_*median*_) and region-of interest (ROI) averaged (${LSQ}_{mean}^{ROI}$ and ${LSQ}_{median}^{ROI}$) magnitude derived estimates. Note the logarithmic y-scale of the *D* ^∗^ estimates plot. Middle column: parameter estimate variability is displayed as 3.9 × prior standard deviations from the BSP estimation ($3.9\times {\varSigma}_{pp}^{1/2},p=d,f$and *d* ^∗^), as width of the 95% interval of the LSQ estimates and 3.9 × standard deviation over each region-of-interest of the LSQ estimates (scaled by 3.9 to approximate the 95% interval assuming a Gaussian distribution). Right column: parameter uncertainty displayed as median of estimated standard deviation under the posterior distribution (*σ*_*p*_, *p* = *d*, *f* and *d* ^∗^)

Figure 6 summarizes various parameter estimates together with regression quality measures (LSQ red boxes, BSP blue boxes) as in [30]. The left column summarizes mean and median estimates across the corresponding regions-of-interest of all parameters. Both the LSQ mean and median estimates of *D* tend to relatively high values compared to BSP, while the prior mean of BSP is within the range of previously reported values [20, 40]. Considering the parameter *F*, there are notable differences among the mean and median estimates, indicating the presence of fitting outliers. Again, the prior mean of BSP is in the range of previously reported values [20]. The mean estimates of *D* ^∗^ are strongly influenced by (high valued) outliers in the region-of-interest, explaining the difference between mean and median LSQ estimates. The BSP prior means take values close to the LSQ median values. The variability measures within the region-of-interest in the middle column show reduced variability for BSP versus LSQ in all parameters both considering standard deviation and percentile based measures. The fit quality in terms of median estimated standard deviation under the posterior (for example *σ*_*d*_) is displayed in the right column. The BSP based deviations are consistently lower compared to the LSQ based values.

**Fig. 7:**
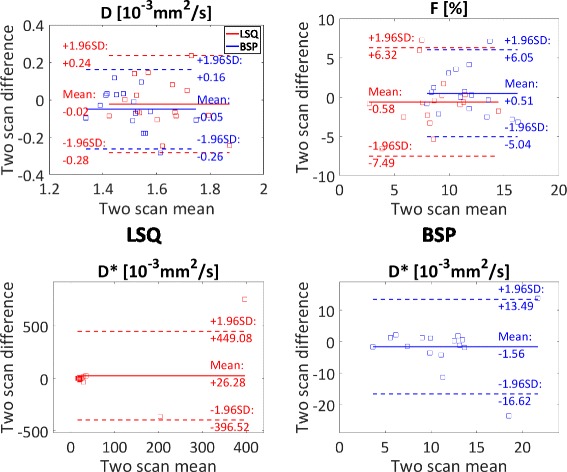
Bland-Altman analysis. Bland-Altman plots showing intra-subject reproducibility of the medians across the regions-of-interest of two consecutive scan sessions for both LSQ and BSP. Medians were chosen to reduce the influence of the high ratio of LSQ outliers. Note the different plot ranges of *D* ^∗^ for LSQ and BSP

For reproducibility analysis, medians across the left ventricular myocardium/region-of-interest were considered because of the large amount of outliers for LSQ fitting. Figure 7 shows the Bland-Altman analysis of two consecutive scans within one session. Mean biases (LSQ/BSP) of −0.02/−0.05·10^−3^ mm^2^/s for *D*, −0.58/+0.51% for *F* and +26.28/−1.56·10^−3^ mm^2^/s for *D* ^∗^ were found. The Bland-Altman coefficients of repeatability are (LSQ/BSP): 0.26/0.21·10^−3^ mm^2^/s for *D*, 6.91/5.55% for F and 422.80/15.06·10^−3^ mm^2^/s for D*.

**Fig. 8:**
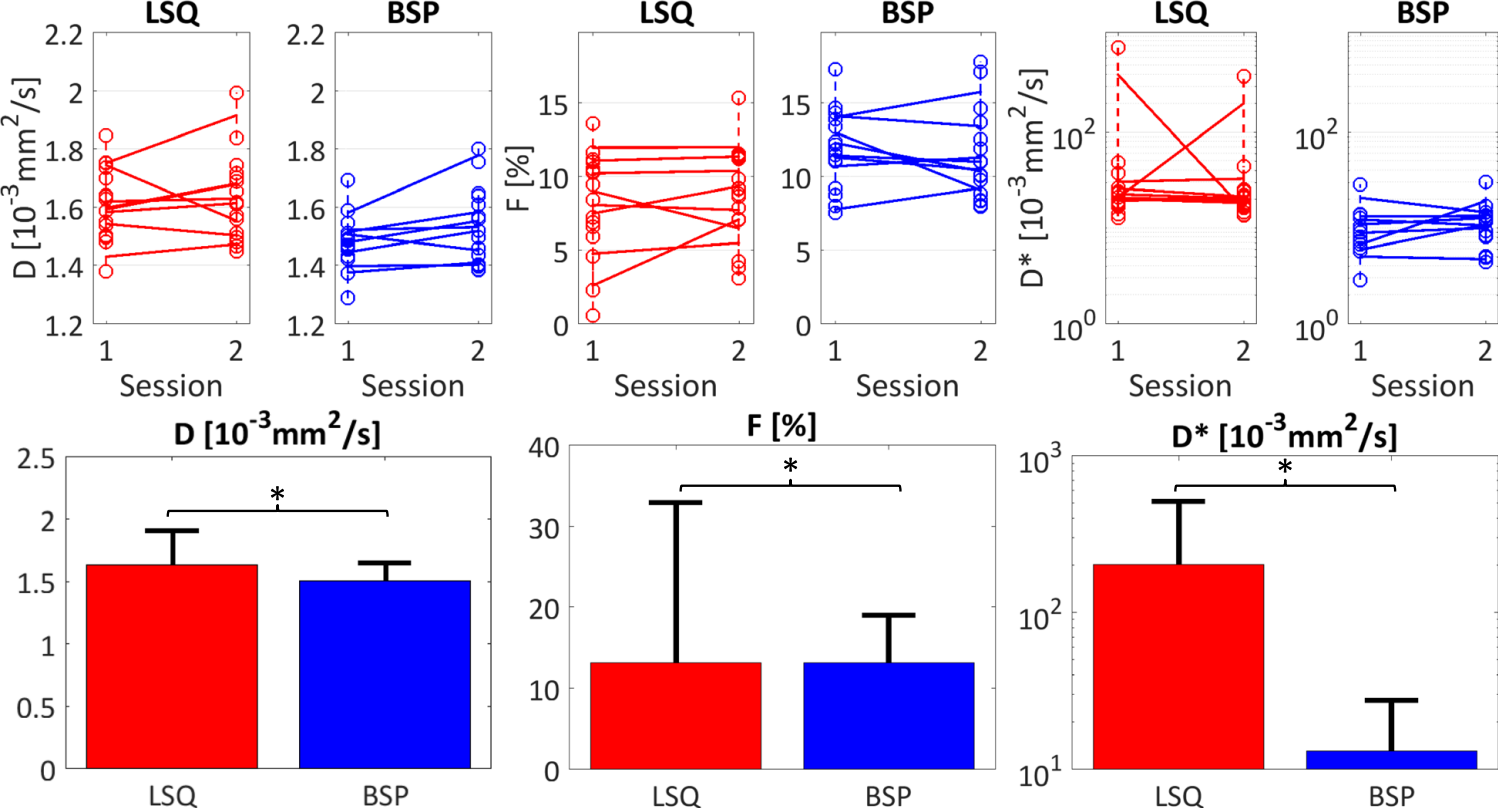
Summary. Top row: medians across the regions-of-interest for the two sessions and scans (connected by dashed lines). The means of the medians of the two intra-session repetitions are connected by solid lines for the two sessions. The BSP estimates are clustered closer together and cover a smaller range compared to the LSQ derived estimates. Bottom row: means and standard deviations of all estimates across all volunteers and repetitions. The standard deviations are consistently smaller for BSP. The parameter estimates of the two methods are significantly different (*, *p* < 0.05). Note the logarithmic y-axes for *D* ^∗^

Figure 8 shows a summary over all measurement estimates. The upper row displays medians across the regions-of-interest for both sessions. As in Fig. 6, the estimates of *D* were found to be higher for LSQ compared to BSP. The medians of the LSQ/BSP estimates are covering ranges of 0.61/0.51·10^−3^ mm^2^/s for *D*, 14.79/10.27% for *F*, 763.37/27.42·10^−3^ mm^2^/s for *D* ^∗^. The lower row of Fig. 8 reports all measurements by displaying the means across all voxels within the regions-of-interest and the corresponding standard deviations. The mean values of the IVIM parameters are (LSQ/BSP) 1.63 ± 0.28/1.51 ± 0.14·10^−3^ mm^2^/s for *D*, 13.13 ± 19.81/13.11 ± 5.95% for *F* and 201.45 ± 313.23/13.11 ± 14.53·10^−3^ mm^2^/s for *D* ^∗^. The estimates for all IVIM parameters from the two inference procedures are significantly different (*p* < 0.05) from each other using the Wilcoxon signed-rank test. Both mean *D* and *F* are within 10% relative difference, but the mean estimates of *D* ^∗^ are one order of magnitude different from each other. This is again due to the high number of outliers produced by the LSQ method. The standard deviations of all three IVIM parameters are consistently lower for BSP compared to LSQ.

**Fig. 9:**
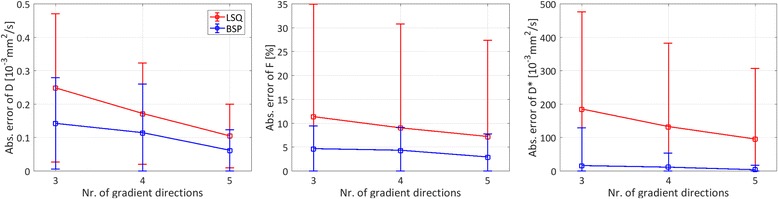
Data subsampling. Absolute pixel-wise parameter estimation error for both LSQ and BSP methods versus number of used diffusion encoding gradient directions. Boxes indicate mean values across all voxels of all measurements; error bars are displaying corresponding standard deviations

Potential scan time reduction was investigated by retrospectively skipping diffusion encoding gradient directions. The reduced SNR due to data subsampling leads to a mean absolute error across all voxels of all measurements of 0.25/0.14·10^−3^ mm^2^/s (*D*), 11.38/4.67% (*F*) and 185.38/15.82·10^−3^ mm^2^/s (*D* ^∗^) for LSQ/BSP if only three instead of all six directions are used as shown in Fig. 9.

**Fig. 10:**
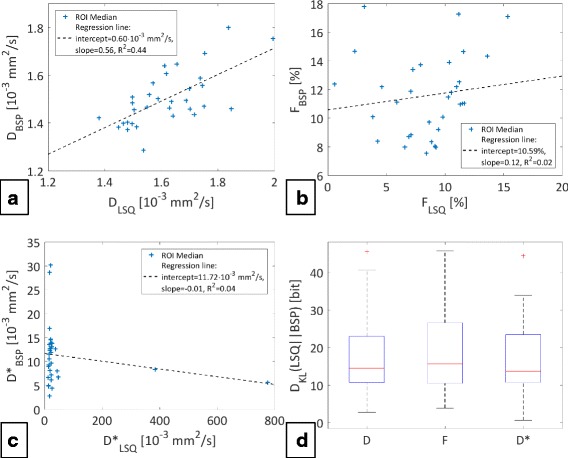
Regression lines & Kullback-Leibler divergences. The plots **a**, **b** and **c** display the medians across the corresponding regions-of-interest (ROI) of all measurements together with regression lines. Respective coefficients are shown in the legends. The plot **d** summarizes the Kullback-Leibler divergences of the BSP estimates from the LSQ estimates of all measurements

The LSQ and BSP estimates were further compared by a regression analysis of the median estimates across the corresponding regions-of-interest as shown in Fig. 10. The diffusion coefficient exhibits an approximately linear correlation between the two methods with LSQ estimates tending to higher values. For the perfusion parameters there is no clear linear correlation. Especially *D* ^∗^ exhibits outliers. The Kullback-Leibler divergences *D*_*KL*_(*LSQ*| | *BSP*) of the BSP parameter estimates from the LSQ estimates are summarized for all measurements in Fig. 10. The median divergences and standard deviations were: 15 ± 11/16 ± 11/14 ± 10 bit for *D*/*F*/*D* ^∗^ respectively.

The infarcted septal region in an animal model exhibits reduced blood flow in a conventional contrast enhanced first pass perfusion scan as well as reduced IVIM perfusion parameters for both regression methods. The BSP derived maps do not contain outliers and hence allow a clearer delineation of the infarcted area compared to LSQ. Further details can be found in Appendix 2.

## Discussion

In the present work, Bayesian shrinkage prior inference has been implemented and compared to segmented least-squares fitting for IVIM parameter mapping in the in-vivo human heart.

Robust data acquisition was possible using a second-order motion-compensated diffusion weighted spin-echo sequence [17] triggered to early systole. Using a trigger delay of 25% peak systole is advantageous because of increased coronary flow compared to peak contraction. Moreover, a large part of systole is potentially available for IVIM acquisitions as shown in [17]: trigger delays in the range of 15–77/79% peak systole at the apex/base allow for robust diffusion data acquisition. In addition, imaging in systole has the advantage of a relatively thick myocardium compared to the voxel size.

Using motion-compensated diffusion gradients may lead to a reduced sensitivity to blood circulation and myocardial perfusion [41]. However, deviations from a mono-exponential diffusion model at lower b-values due to perfusion were observed in all measurements indicating sufficient sensitivity to microcirculation and perfusion. Balancing motion-induced signal loss due to cardiac bulk motion while achieving optimal sensitivity to perfusion is however a subject deserving further attention.

Computer simulations revealed minimum SNR thresholds of 30–60 for relative errors in terms of bias and variation of 20% each, depending on the perfusion regime for BSP while the LSQ method required a minimum SNR of at least 200. The increase in bias in *F* for the SNR range of 125–175 using LSQ is suspected to be an artefact of the segmented fit, which leads to an error propagation of *D* and *S*_*int*_ into the estimation of the perfusion parameters. Furthermore, we note that Federau and colleagues also reported a similar increase in bias in *F* in Fig. 1 of [27] using a segmented approach; albeit in the SNR range of approx. 20–40 with simulated IVIM parameters which are commonly found in the brain (*D*=0.7·10^−3^mm^2^s, *F*=4% and *D* ^∗^=17·10^−3^mm^2^s). Even though the relative bias in *F* in the considered SNR range is below about 20%, the segmented fit might benefit from a joint parameter estimation (potentially using two regimes of mono- and bi-exponential decay) in this regard. While these simulation results are indicative, several factors confounding in-vivo measurements have not been taken into account in the simulations including residual motion artifacts and partial voluming with hyperintense blood signal and epicardial fat [42]. These effects would lead to a broadening of the parameter histograms. Accordingly, the width of the prior is increased by the presence of a large number of affected voxels. The shrinking procedure in these cases is less effective.

In-vivo, BSP analysis resulted in IVIM parameter maps with considerably smaller intra-myocardial standard deviations relative to LSQ. Both variability and estimation uncertainty in terms of standard deviation under the posterior were greatly reduced with BSP compared to LSQ (Fig. 6), indicating the benefit of taking into account prior knowledge. The setting of arbitrary fit constraints was obsolete in the BSP procedure. In addition, BSP regression was aided by the prior which led to the elimination of outliers on or close to the fit boundaries.

An effective spatial denoising of the parameters can be achieved because the prior in BSP is chosen to be a unimodal distribution. This prior assumes a population mean of the IVIM parameters for the whole region-of-interest and hence assumes the myocardium of the left ventricle to have a rather homogenous spatial tissue characteristic. If local pathologies such as myocardial infarcts (as for example presented in Appendix 2) and corresponding fibrous tissue with reduced perfusion [14] are present, a different choice of priors such as a spatial homogeneous prior [43] is deemed more appropriate [42]. Alternatively, multimodal priors can be applied to distinguish among different tissue types while still retaining spatial information. Methods using mixture models [44] of multivariate Gaussians could also be implemented to address this limitation. In contrast to the LSQ approach, which allows data processing on a pixel-by-pixel basis, the BSP method requires pre-segmentation of the data, which renders automation in a post-processing workflow more challenging.

Overall, the in-vivo IVIM parameters measured in this study are in good accordance with recent literature [10, 20]. The diffusion coefficients *D* found in this study using LSQ and BSP were within the range of the values found in [10, 20]. A higher measured diffusivity is indicative of residual motion effects in the data [45]. In addition, the mean diffusivity measured using spin-echo based diffusion tensor imaging (DTI) during early systole [40] was within 14% and 6% of the mean measured diffusion coefficient of the present study for LSQ and BSP, respectively. The measured perfusion fraction *F* was found to be lower compared to data reported in [10]. The LSQ and BSP estimation of approx. 13% is close to the 12% found in [20] during diastole. The pseudo-diffusion coefficients *D* ^∗^ of 201.50 ± 313.20 mm^2^/s (LSQ) and 13.11 ± 14.53 mm^2^/s (BSP) as measured in the present study are different from previous data (52.68 ± 52.61·10^−3^ mm^2^/s [10], 43.6 ± 9.2·10^−3^ mm^2^/s [20]). However, *D* ^∗^ usually contains the highest number of outliers and the mean across the region-of-interest is therefore prone to be heavily influenced by the choice of the actual value of the box-constraints. If the medians across the regions-of-interest are considered, it is shown that the majority of the LSQ estimates gather in the range of 20 to 50·10^−3^ mm^2^/s. Moreover, data in Fig. 6 indicates that mean estimates of *D* ^∗^ are considerably influenced by outliers with median parameter values close to the ones found using the BSP method.

The IVIM perfusion parameters in an infarcted animal model (see also Appendix 2) show good accordance with the perfusion defect visible in the contrast enhanced perfusion scan, especially considering the blood flow related [25, 27] product of *F* × *D* ^∗^ of the BSP derived estimates. However, the extension of the infarct indicated by IVIM appears smaller compared to the darkened area of the contrast enhanced perfusion scan. This might be due to residual motion and/or partial voluming which can yield elevated IVIM parameter estimates.

All SNR measurements were obtained from a single signal average. Thereby confounding factors due to image registration and phase correction for averaging of complex data were avoided. The in-vivo SNR was above the 20% parameter error threshold found in simulations. The scan time of ca. 18 min for a heart rate of 60 bpm for this study was in-between previous scanning times of 15 min [10] and 20 min [20]. Optimizations of the experiment design in terms of b-value distribution [46], higher static field strength and improved gradient performance to reduce echo times may allow to reduce parameter estimation error and scan time.

By design, Bayesian approaches exploiting information across the region-of-interest can be used to examine distributed rather than focal pathologies of the myocardium. Accordingly, potential applications relate to microvascular obstruction and reduced and/or delayed perfusion of the myocardium in hypertrophic cardiomyopathy and diabetes [15, 47].

## Conclusion

Bayesian IVIM parameter mapping yields improved parameter maps relative to conventional segmented least-squares fitting in the human heart. In conjunction with motion-compensated diffusion weighted spin-echo sequences, robust parameter estimation can be achieved providing a tissue perfusion surrogate without contrast agent application. Further in-vivo studies are now warranted to assess the performance of the method in relevant patient populations.

## Figures and Tables

**Fig. 11:**
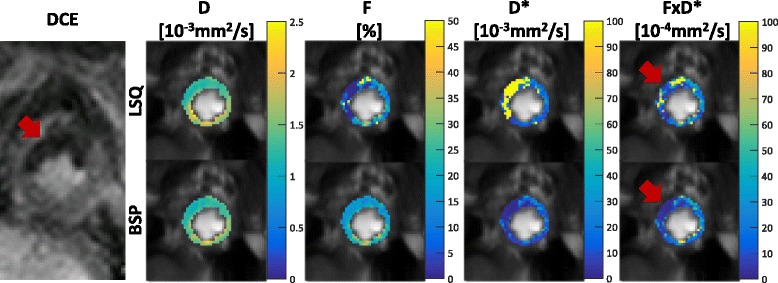
Infarcted porcine heart animal sub study. Dynamic contrast enhanced (DCE) first pass imaging shows a myocardial infarct in the septal area (red arrow). Corresponding IVIM parameter maps in the same slice position reveal a reduction in perfusion related parameters in the septal region. The LSQ approach suffers from many outliers, while the BSP approach shows low parameter variability (red arrows)

## Appendix 1

### Hierarchical Bayesian modelling

The Bayesian inference procedure [30] is summarized as follows. Using the IVIM model

5 $$ {S}_n={S}_0\left[F\cdot \exp \left(-{b}_n{D}^{\ast}\right)+\left(1-F\right)\cdot \exp \left(-{b}_nD\right)\right]+{\varepsilon}_n\kern0.5em , $$

where *S*_*n*_ is a data point measured at the n-th b-value *b*_*n*_ with error term *ε*_*n*_ of Gaussian distribution with variance *σ*_*S*_^2^, the data likelihood reads:

6 $$ p\left(\boldsymbol{S}|D,F,{D}^{\ast };{S}_0,{\sigma_S}^2\right)={\left(2{{\pi \sigma}_S}^2\right)}^{-N/2}\exp \left(-\frac{1}{2{\sigma_S}^2}\sum \limits_{n=1}^N\left({S}_n-{S}_0{g}_n\right)\right)\kern0.5em , $$

where ***S*** = [***S***_1_,  ***S***_2_, …***S***_***N***_]^***T***^ **,** *g*_*n*_ = *F* ∙ exp(−*b*_*n*_ *D* ^∗^) + (1 − *F*) ∙ exp(−*b*_*n*_ *D*) and *N*=number of b-values.

From a Bayesian perspective, nuisance parameters which are of no interest can be marginalized out. Here, the nuisance parameters *S*_0_ and *σ*_*S*_^2^ are integrated out by using a conjugate Normal-Inverse-Gamma prior distribution

7 $$ p\left({S}_0,{\sigma_S}^2\right)=N\left({S}_0|0,{\delta}^2{\sigma_S}^2/\left({\boldsymbol{g}}^T\boldsymbol{g}\right)\right)\cdot IG\left({\sigma_S}^2|\alpha, \beta \right) $$

with ***g*** = [***g***_1_,  ***g***_2_, …***g***_***N***_]^***T***^ and integration over the domain of definition:

8 $$ p\left(\boldsymbol{S}|D,F,{D}^{\ast}\right)=\underset{0}{\overset{\infty }{\int }}\underset{-\infty }{\overset{\infty }{\int }}p\left({S}_0,{\sigma_S}^2\right)p\left(\boldsymbol{S}|D,F,{D}^{\ast };{S}_0,{\sigma_S}^2\right)\ {dS}_0\ d{\sigma_S}^2 $$

The choice of prior allows for an analytic evaluation of Eq. (8). The influence of the prior for the marginalization procedure is diminished by taking the limits of the variance of the normal distribution *δ* → ∞ and shape and scale parameters of the Inverse Gamma distribution *α*, *β* → 0, which encodes a complete lack of prior information. Ignoring proportionality constants which do not depend on the IVIM parameters and are hence not necessary for parameter inference, the marginalized likelihood becomes:

9 $$ p\left(\boldsymbol{S}|D,F,{D}^{\ast}\right)\propto {\left[{\boldsymbol{S}}^T\boldsymbol{S}-{\left({\boldsymbol{S}}^T\boldsymbol{g}\right)}^2/\left({\boldsymbol{g}}^T\boldsymbol{g}\right)\right]}^{-N/2} $$

In order to take advantage of the histogram structure of IVIM parameters, a hierarchical prior structure is adopted. First, a multivariate Gaussian is applied to the transformed IVIM parameters. Those transformations are mapping the domain of definition to the field of real numbers:

10 $$ {\displaystyle \begin{array}{c}\ d=\log (D)\\ {}f=\mathrm{logit}(F)=\log (F)-\log \left(1-F\right)\\ {}{d}^{\ast }=\log \left({D}^{\ast}\right)\end{array}} $$

The multivariate normal distribution considers heterogeneity across the region-of-interest and models correlations between the parameters via the covariance matrix *Σ*_*μ*_:

11 $$ p\left({\boldsymbol{\theta}}_i|\boldsymbol{\mu}, {\varSigma}_{\mu}\right)={\left|2\pi {\varSigma}_{\mu}\right|}^{-1/2}\exp \left(-\frac{1}{2}{\left({\boldsymbol{\theta}}_i-\boldsymbol{\mu} \right)}^T{\varSigma_{\mu}}^{-1}\left({\boldsymbol{\theta}}_i-\boldsymbol{\mu} \right)\right) $$

The mean across the region-of-interest of the parameter ***θ***_*i*_ = [*d*_*i*_, *f*_*i*_, *d* ^∗^_*i*_] of voxel *i* is $\boldsymbol{\mu} =\left[{\mu}_d,{\mu}_f,{\mu}_{d^{\ast }}\right]$. Jeffrey’s prior [31] is used for the hyper-parameters ***μ*** and *Σ*_*μ*_:

12 $$ p\left(\boldsymbol{\mu}, {\varSigma}_{\mu}\right)={\left|I\left({\varSigma}_{\mu}\right)\right|}^{1/2}={\left|{\varSigma}_{\mu}\right|}^{-1/2} $$

This prior describes a high probability for a large determinant of the Fisher Information *I*(*Σ*_*μ*_) and hence in the considered case of a multivariate normal distribution for a small determinant of the parameter covariance matrix *Σ*_*μ*_ in the region-of-interest. Therefore a “shrinking” of parameter estimates towards the mean of the distribution can typically be observed. The posterior is then given by

13 $$ p\left({\boldsymbol{\theta}}_{1:M},\boldsymbol{\mu}, {\varSigma}_{\mu }|{\boldsymbol{S}}_{1:M}\right)=\frac{p\left(\boldsymbol{\mu}, {\varSigma}_{\mu}\right){\prod}_{i=1}^Mp\left({\boldsymbol{S}}_i|{\boldsymbol{\theta}}_i\right)p\left({\boldsymbol{\theta}}_i\right)}{p\left({\boldsymbol{S}}_{1:M}\right)} $$

The total number of voxels considered here is *M*. The parameter independent data evidence *p*(***S***_1 : *M*_), is not required for the inference procedure. Expectation values under the posterior in Eq. (13) are calculated for example as

14 $$ {\widehat{d}}_i=\int {d}_ip\left({\boldsymbol{\theta}}_{i:M},\boldsymbol{\mu}, {\varSigma}_{\mu }|{\boldsymbol{S}}_{1:M}\right)\ d{\boldsymbol{\theta}}_{i:M}\ d{\varSigma}_{\mu }\ d\boldsymbol{\mu} $$

and analogously for other parameters or quantities.

In order to determine variance under the posterior as uncertainty measure for the LSQ method, a flat prior according to the box constraints in Eq. (4) is chosen instead of the hierarchical prior structure [30].

#### Markov chain Monte Carlo (MCMC) implementation

The integration in Eq. (14) cannot be performed analytically and therefore a MCMC-based numerical approach is implemented [30]. The expectation value of a parameter (for example *d*) is approximated by using *N*_*s*_ samples ${d}_i^{(j)}$from a Markov chain output:

15 $$ {\widehat{d}}_i=\frac{1}{N_s}\sum \limits_{j=1}^{N_s}{d_i}^{(j)} $$

## Appendix 2

### Infarcted porcine heart

Both LSQ and BSP approaches were compared in an animal model of acute myocardial infarction. Obtained diffusion weighted data from a single female pig (65 kg, heart-rate 68 beats/min) using second-order motion-compensated diffusion gradients [17] were evaluated. The imaging parameters were: in-plane resolution: 2.4 × 2.4 mm^2^, slice thickness: 10 mm, one apical slice, FOV: 230 × 120 mm^2^, acquired k-space lines: 49, TR/TE: 2 R-R/93 ms, trigger delay 50% peak systole, flip angle: 90°, 8 signal averages and 6 diffusion encoding directions [48]. The 15 optimized diffusion encoding strengths were taken from [46] with a maximum b-value of 740 s/mm^2^. The apical myocardial infarct was induced by a permanent distal ligation of the left anterior descending coronary artery (LAD). The animal was anesthetized by a constant dose of Propofol (1.0 ml/kg/min) during surgery and the scan. The experiment was performed in adherence to the Swiss law of animal protection and approved by the Zurich Cantonal veterinary office.

The IVIM parameter maps are shown together with dynamic contrast enhanced (DCE) first pass perfusion images in Fig. 11. The infarcted area can be delineated in the septal area (red arrow). The LSQ and BSP derived diffusion coefficients *D* are similar across the whole LV. The perfusion fraction *F* is reduced in the septal area for both methods, with outliers present for LSQ. The pseudo-diffusion coefficient *D* ^∗^ is also clearly reduced in this area for BSP but shows insensible high values for LSQ due to the optimization reaching the fit constraints. If the blood flow related [25, 27] product of *F* × *D* ^∗^ is considered, the LSQ derived product does not allow to delineate the infarcted region due to many outliers. In contrast, the septal region in the BSP derived map shows reduced IVIM perfusion parameters.

## Abbreviations

BSP: Bayesian shrinkage prior
FOV: Field-of-view
IVIM: Intravoxel incoherent motion
LoLo: Local-look
LSQ: Least-squares
MCMC: Markov chain Monte Carlo
PCA: Principal component analysis
SNR: Signal-to-noise ratio
TE: Echo time
TR: Repetition time
